# Supplementary material for: Neighborhood Disadvantage and Tobacco Retail Outlet and Vape Shop Outlet Rates
Source: Int J Environ Res Public Health. 2020 Apr 21;17(8):2864. doi: 10.3390/ijerph17082864 (PMC7215286; doi:10.3390/ijerph17082864)
Supplement: Supplementary file 1 [file ijerph-17-02864-s001.pdf]

Neighborhood Disadvantage and Tobacco Retail Outlet and Vape Shop Outlet Rates

David C. Wheeler [1,2,*], Elizabeth K. Do [2,3], Rashelle B. Hayes [2,4], Kendall Fugate-Laus [3], Westley L. Fallavollita [3], Colleen Hughes [5] and Bernard F. Fuemmeler [2,3]

[1] Department of Biostatistics, Virginia Commonwealth University, Richmond, Virginia, USA
[2] Massey Cancer Center, Virginia Commonwealth University, Richmond, Virginia, USA
[3] Department of Health Behavior & Policy, Virginia Commonwealth University, Richmond, Virginia, USA
[4] Department of Psychiatry, Virginia Commonwealth University, Richmond, Virginia, USA
[5] Department of Behavioral Health & Developmental Services, Richmond, Virginia, USA
* Correspondence: david.wheeler@vcuhealth.org

Table S1. Census tracts with significantly elevated relative risk for tobacco retail outlets

FIPS	County	Census Tract
51001	Accomack County	Census Tract 901
51001	Accomack County	Census Tract 902
51001	Accomack County	Census Tract 904
51001	Accomack County	Census Tract 908
51001	Accomack County	Census Tract 906
51001	Accomack County	Census Tract 907
51009	Amherst County	Census Tract 105.04
51011	Appomattox County	Census Tract 401
51013	Arlington County	Census Tract 1036.02
51015	Augusta County	Census Tract 709
51015	Augusta County	Census Tract 701
51017	Bath County	Census Tract 9201
51025	Brunswick County	Census Tract 9302.03
51025	Brunswick County	Census Tract 9302.01
51027	Buchanan County	Census Tract 102
51027	Buchanan County	Census Tract 101
51027	Buchanan County	Census Tract 106
51031	Campbell County	Census Tract 209
51031	Campbell County	Census Tract 207
51035	Carroll County	Census Tract 806.02
51035	Carroll County	Census Tract 801
51035	Carroll County	Census Tract 802
51037	Charlotte County	Census Tract 9301
51037	Charlotte County	Census Tract 9302
51037	Charlotte County	Census Tract 9303
51041	Chesterfield County	Census Tract 1003
51051	Dickenson County	Census Tract 402
51051	Dickenson County	Census Tract 403
51051	Dickenson County	Census Tract 401

51057	Essex County	Census Tract 9507
51059	Fairfax County	Census Tract 4516.01
51059	Fairfax County	Census Tract 4802.02
51059	Fairfax County	Census Tract 4525.01
51059	Fairfax County	Census Tract 4520
51063	Floyd County	Census Tract 9201.02
51067	Franklin County	Census Tract 208
51067	Franklin County	Census Tract 202
51071	Giles County	Census Tract 9303
51073	Gloucester County	Census Tract 1003.01
51077	Grayson County	Census Tract 602.01
51081	Greensville County	Census Tract 8802
51083	Halifax County	Census Tract 9306
51083	Halifax County	Census Tract 9302.01
51083	Halifax County	Census Tract 9308
51083	Halifax County	Census Tract 9304
51085	Hanover County	Census Tract 3206.02
51085	Hanover County	Census Tract 3206.01
51087	Henrico County	Census Tract 2008.02
51089	Henry County	Census Tract 106.02
51089	Henry County	Census Tract 106.01
51089	Henry County	Census Tract 113
51089	Henry County	Census Tract 103
51089	Henry County	Census Tract 101
51089	Henry County	Census Tract 110
51089	Henry County	Census Tract 111
51089	Henry County	Census Tract 102
51089	Henry County	Census Tract 112
51091	Highland County	Census Tract 9701
51093	Isle of Wight County	Census Tract 2801.05
51093	Isle of Wight County	Census Tract 2803
51099	King George County	Census Tract 401
51103	Lancaster County	Census Tract 302
51105	Lee County	Census Tract 9503
51107	Loudoun County	Census Tract 6105.05
51107	Loudoun County	Census Tract 6117
51115	Mathews County	Census Tract 9514
51117	Mecklenburg County	Census Tract 9301.01
51117	Mecklenburg County	Census Tract 9302
51117	Mecklenburg County	Census Tract 9306
51117	Mecklenburg County	Census Tract 9304
51119	Middlesex County	Census Tract 9510
51121	Montgomery County	Census Tract 207

51121	Montgomery County	Census Tract 203
51121	Montgomery County	Census Tract 214
51121	Montgomery County	Census Tract 208
51125	Nelson County	Census Tract 9503
51131	Northampton County	Census Tract 9301
51131	Northampton County	Census Tract 9303
51131	Northampton County	Census Tract 9302
51133	Northumberland County	Census Tract 201
51135	Nottoway County	Census Tract 3
51135	Nottoway County	Census Tract 1
51141	Patrick County	Census Tract 303.01
51141	Patrick County	Census Tract 302
51141	Patrick County	Census Tract 301
51143	Pittsylvania County	Census Tract 108.02
51143	Pittsylvania County	Census Tract 110.01
51143	Pittsylvania County	Census Tract 106
51143	Pittsylvania County	Census Tract 105
51147	Prince Edward County	Census Tract 9301
51147	Prince Edward County	Census Tract 9302.01
51153	Prince William County	Census Tract 9004.03
51153	Prince William County	Census Tract 9014.03
51153	Prince William County	Census Tract 9006
51153	Prince William County	Census Tract 9017.01
51153	Prince William County	Census Tract 9019
51153	Prince William County	Census Tract 9002.01
51155	Pulaski County	Census Tract 2102.01
51155	Pulaski County	Census Tract 2101
51155	Pulaski County	Census Tract 2106
51159	Richmond County	Census Tract 401
51161	Roanoke County	Census Tract 302.04
51163	Rockbridge County	Census Tract 9304
51163	Rockbridge County	Census Tract 9301
51165	Rockingham County	Census Tract 102
51167	Russell County	Census Tract 305
51169	Scott County	Census Tract 301
51169	Scott County	Census Tract 306
51171	Shenandoah County	Census Tract 407
51171	Shenandoah County	Census Tract 408
51171	Shenandoah County	Census Tract 405
51173	Smyth County	Census Tract 302
51173	Smyth County	Census Tract 307.01
51173	Smyth County	Census Tract 305
51175	Southampton County	Census Tract 2004

51177	Spotsylvania County	Census Tract 203.04
51177	Spotsylvania County	Census Tract 201.08
51177	Spotsylvania County	Census Tract 203.05
51183	Sussex County	Census Tract 8703
51183	Sussex County	Census Tract 8704
51183	Sussex County	Census Tract 8701
51185	Tazewell County	Census Tract 210
51185	Tazewell County	Census Tract 211
51187	Warren County	Census Tract 204
51187	Warren County	Census Tract 205
51191	Washington County	Census Tract 109
51191	Washington County	Census Tract 107
51191	Washington County	Census Tract 104.02
51191	Washington County	Census Tract 105.02
51191	Washington County	Census Tract 106.01
51193	Westmoreland County	Census Tract 102
51195	Wise County	Census Tract 9308
51195	Wise County	Census Tract 9315
51195	Wise County	Census Tract 9317
51195	Wise County	Census Tract 9312
51195	Wise County	Census Tract 9309
51197	Wythe County	Census Tract 504.02
51197	Wythe County	Census Tract 501
51197	Wythe County	Census Tract 502
51510	Alexandria City	Census Tract 2012.03
51520	Bristol City	Census Tract 204
51520	Bristol City	Census Tract 201
51520	Bristol City	Census Tract 202
51530	Buena Vista City	Census Tract 9306
51540	Charlottesville City	Census Tract 10
51540	Charlottesville City	Census Tract 8
51550	Chesapeake City	Census Tract 207
51550	Chesapeake City	Census Tract 203
51550	Chesapeake City	Census Tract 209.06
51550	Chesapeake City	Census Tract 211.01
51550	Chesapeake City	Census Tract 210.04
51550	Chesapeake City	Census Tract 208.05
51550	Chesapeake City	Census Tract 204
51570	Colonial Heights City	Census Tract 8301
51580	Covington City	Census Tract 602
51590	Danville City	Census Tract 2
51590	Danville City	Census Tract 6
51590	Danville City	Census Tract 4

51590	Danville City	Census Tract 13.01
51590	Danville City	Census Tract 13.02
51590	Danville City	Census Tract 5
51590	Danville City	Census Tract 14
51590	Danville City	Census Tract 7
51590	Danville City	Census Tract 12
51590	Danville City	Census Tract 8
51590	Danville City	Census Tract 1
51590	Danville City	Census Tract 9
51595	Emporia City	Census Tract 8902
51595	Emporia City	Census Tract 8901
51600	Fairfax City	Census Tract 3004
51620	Franklin City	Census Tract 901
51620	Franklin City	Census Tract 902
51630	Fredericksburg City	Census Tract 5
51640	Galax City	Census Tract 701.01
51650	Hampton City	Census Tract 105.02
51660	Harrisonburg City	Census Tract 2.03
51660	Harrisonburg City	Census Tract 2.05
51660	Harrisonburg City	Census Tract 4.02
51660	Harrisonburg City	Census Tract 3.02
51660	Harrisonburg City	Census Tract 2.07
51670	Hopewell City	Census Tract 8203
51670	Hopewell City	Census Tract 8206
51680	Lynchburg City	Census Tract 7
51683	Manassas City	Census Tract 9103.01
51683	Manassas City	Census Tract 9102.01
51690	Martinsville City	Census Tract 2
51690	Martinsville City	Census Tract 1
51690	Martinsville City	Census Tract 3
51690	Martinsville City	Census Tract 4
51700	Newport News City	Census Tract 316.02
51700	Newport News City	Census Tract 320.06
51710	Norfolk City	Census Tract 27
51710	Norfolk City	Census Tract 37
51710	Norfolk City	Census Tract 42
51710	Norfolk City	Census Tract 57.01
51710	Norfolk City	Census Tract 69.01
51720	Norton City	Census Tract 9601
51730	Petersburg City	Census Tract 8112
51730	Petersburg City	Census Tract 8103
51730	Petersburg City	Census Tract 8101
51740	Portsmouth City	Census Tract 2115

51740	Portsmouth City	Census Tract 2121
51740	Portsmouth City	Census Tract 2109
51740	Portsmouth City	Census Tract 2123
51740	Portsmouth City	Census Tract 2111
51740	Portsmouth City	Census Tract 2126
51740	Portsmouth City	Census Tract 2128.02
51740	Portsmouth City	Census Tract 2124
51750	Radford City	Census Tract 101.01
51770	Norfolk City	Census Tract 10
51770	Norfolk City	Census Tract 11
51770	Norfolk City	Census Tract 25
51770	Norfolk City	Census Tract 30
51770	Norfolk City	Census Tract 3
51770	Norfolk City	Census Tract 5
51770	Norfolk City	Census Tract 9
51770	Norfolk City	Census Tract 26
51770	Norfolk City	Census Tract 6.02
51775	Salem City	Census Tract 103
51790	Staunton City	Census Tract 6
51800	Suffolk City	Census Tract 757.01
51800	Suffolk City	Census Tract 754.01
51800	Suffolk City	Census Tract 653
51800	Suffolk City	Census Tract 654
51800	Suffolk City	Census Tract 655
51800	Suffolk City	Census Tract 757.02
51810	Virginia Beach City	Census Tract 426
51810	Virginia Beach City	Census Tract 440.04
51810	Virginia Beach City	Census Tract 440.03
51810	Virginia Beach City	Census Tract 402
51810	Virginia Beach City	Census Tract 442
51810	Virginia Beach City	Census Tract 404.02
51810	Virginia Beach City	Census Tract 448.08
51810	Virginia Beach City	Census Tract 410.02
51810	Virginia Beach City	Census Tract 462.13
51810	Virginia Beach City	Census Tract 458.01
51820	Waynesboro City	Census Tract 31
51820	Waynesboro City	Census Tract 35

Table S2. Census tracts with significantly elevated relative risk for vape shop outlets

FIPS	County Name	Census Tract
51153	Prince William County	Census Tract 9019
51161	Roanoke County	Census Tract 302.04
51187	Warren County	Census Tract 204
51197	Wythe County	Census Tract 502
51520	Bristol City	Census Tract 204
51540	Charlottesville City	Census Tract 10
51550	Chesapeake City	Census Tract 215.02
51550	Chesapeake City	Census Tract 208.05
51570	Colonial Heights City	Census Tract 8301
51590	Danville City	Census Tract 8
51630	Fredericksburg City	Census Tract 5
51660	Harrisonburg City	Census Tract 2.07
51710	Norfolk City	Census Tract 49
51710	Norfolk City	Census Tract 57.01
51710	Norfolk City	Census Tract 69.01
51710	Norfolk City	Census Tract 70.02
51720	Norfolk City	Census Tract 9601
51770	Norfolk City	Census Tract 11
51810	Virginia Beach City	Census Tract 440.04
51810	Virginia Beach City	Census Tract 456.04
51810	Virginia Beach City	Census Tract 448.08
51810	Virginia Beach City	Census Tract 410.02
51810	Virginia Beach City	Census Tract 462.13
51810	Virginia Beach City	Census Tract 454.08
51810	Virginia Beach City	Census Tract 458.01
